# Detection of K-complexes in EEG waveform images using faster R-CNN and deep transfer learning

**DOI:** 10.1186/s12911-022-02042-x

**Published:** 2022-11-17

**Authors:** Natheer Khasawneh, Mohammad Fraiwan, Luay Fraiwan

**Affiliations:** 1 Department of Software Engineering, Jordan University of Science and Technology, P.O. Box 3030, Irbid, 22110 Jordan; 2 Department of Computer Engineering, Jordan University of Science and Technology, P.O. Box 3030, Irbid, 22110 Jordan; 3 Department of Electrical and Computer Engineering, Abu Dhabi University, Abu Dhabi, UAE; 4 Department of Biomedical Engineering, Jordan University of Science and Technology, P.O. Box 3030, Irbid, 22110 Jordan

**Keywords:** K-complex, EEG, Faster R-CNN, Deep learning

## Abstract

**Background:**

The electroencephalography (EEG) signal carries important information about the electrical activity of the brain, which may reveal many pathologies. This information is carried in certain waveforms and events, one of which is the K-complex. It is used by neurologists to diagnose neurophysiologic and cognitive disorders as well as sleep studies. Existing detection methods largely depend on tedious, time-consuming, and error-prone manual inspection of the EEG waveform.

**Methods:**

In this paper, a highly accurate K-complex detection system is developed. Based on multiple convolutional neural network (CNN) feature extraction backbones and EEG waveform images, a regions with faster regions with convolutional neural networks (Faster R-CNN) detector was designed, trained, and tested. Extensive performance evaluation was performed using four deep transfer learning feature extraction models (AlexNet, ResNet-101, VGG19 and Inceptionv3). The dataset was comprised of 10948 images of EEG waveforms, with the location of the K-complexes included as separate text files containing the bounding boxes information.

**Results:**

The Inceptionv3 and VGG19-based detectors performed consistently high (i.e., up to 99.8% precision and 0.2% miss rate) over different testing scenarios, in which the number of training images was varied from 60% to 80% and the positive overlap threshold was increased from 60% to 90%.

**Conclusions:**

Our automated method appears to be a highly accurate automatic K-complex detection in real-time that can aid practitioners in speedy EEG inspection.

**Graphical Abstract:**

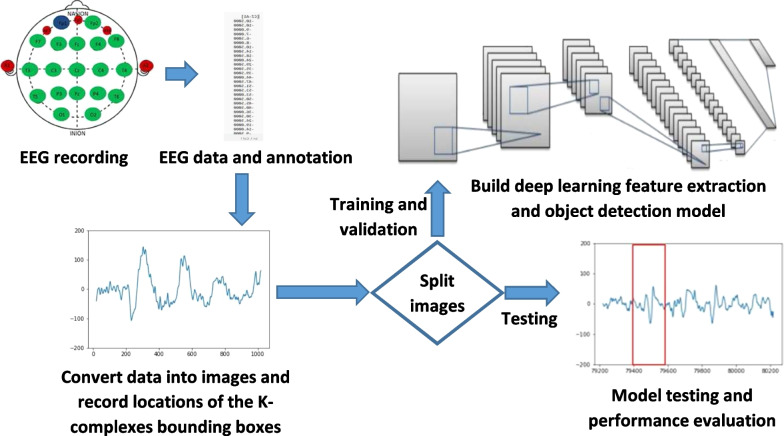

**Supplementary Information:**

The online version contains supplementary material available at 10.1186/s12911-022-02042-x.

## Background

The electroencephalography (EEG) recording shows the scalp’s electrical activity on different locations using several channels. It reveals the electrical activity produced by the brain neurons, which may aid in revealing several pathologies, such as epilepsy, tumors, coma, brain damage, and encephalopathies. EEG analysis has been shown to be a powerful tool for sleep studies and diagnosis of neurological diseases [1]. The EEG exhibits several waveforms such as alpha and beta and events like K-complexes and sleep spindles [2].

The EEG recording is a time-varying continuous signal with an amplitude range of 10–200 μV and a frequency range of 0.5–50 Hz [3]. Detecting the occurrence of the waveform and events is of great importance in clinical practice and considered in many cases to be tedious and time-consuming. This is especially true for long-duration recordings in sleep analysis, where neurologists must evaluate the entire sleep recordings. Therefore, an automated approach for detecting EEG waveforms and events can be very helpful in supporting the clinical decision, because it will shorten the duration of the evaluation and provide a tool to detect the relevant waveforms and events.

Advances in signal processing and artificial intelligence techniques have enabled active research into automated algorithms that facilitate the usability of EEG recordings [4]. To this end, the work in this paper aimed at identifying K-complexes in EEG waveform images using deep transfer learning and faster regions with convolutional neural networks (Faster R-CNN) [5]. K-complexes occur during the non-rapid eye movement (NREM) during sleep stage N2. The transient waveform performed by the K-complex has a biphasic morphology, which has 200 ms waves that are characterized by a positive rise followed by a negative fall of 550 ms with a long-lasting positive peak of 900 ms [6]. Therefore, the presence of K-complexes has an important role in the clinical diagnosis of diseases like Alzheimer’s, insomnia, epilepsy, restless legs syndrome (RLS), and obstructive sleep apnea (OSA) [7, 8].

Several studies in the literature were conducted to detect K-complexes. The pioneering study of Bremer et al. [9] developed a real-time hardware-based automatic K-complex detection system, which can also work offline. However, topical detection methods provide software-based solutions. The majority of these methods are based on signal processing techniques to transform the EEG signal into a more usable form (e.g., segmentation) and extract distinctive features of the K-complexes that can be fed to AI-based classifiers. For example, Noori et al. [10] used chaotic features and the modified extreme learning machine-generalized radial basis function (MELM-GRBF) classifier. Dumitrescu et al. [11] aimed at improving the computation time and detection accuracy using Cohen class recursiveness and reallocation in conjunction with deep learning. Several studies were conducted by Al-Salman et al. [12–14] in which they experimented with several feature extraction techniques for K-complex detection (i.e., multi-domain feature extraction and fractal dimension of time frequency images) in conjunction with a number of classification algorithms (i.e., K-means, least square support vector machine, and Naïve Bayes). Consequently, Kantar and Erdmar [15] used the same procedure. They extracted three features from the EEG records and used support vector machine classifier. Yucelbas et al. [16] investigated the use of the time-frequency analysis of the EEG recordings to detect K-complexes. They used three different time-frequency analysis methods: singular value decomposition, discrete wavelet transform, and variational mode decomposition. Several other works were also conducted in the literature [17–26].

The goal of this work was to get rid of the signal processing steps, explicit feature extraction, and transformations (e.g., spectrograms) along with their companion overhead, inaccuracies, and implementation difficulties. This was accomplished by using deep transfer learning artificial intelligence (AI) algorithms and object detection techniques. The approach we follow is different in that we treat the EEG signal visually as a series of waveform images. These images form the input to a deep learning feature extraction model that feeds an object (i.e., the K-complex) detector, which in turn determines the location of the K-complex via a bounding box overlayed on the waveform.

The remainder of this paper is organized as follows: The materials and methods section explains in detail the dataset and EEG signal images, object detection models, experimental setup, and performance evaluation metrics. Afterwards, The results are presented and discussed. We summarize our findings and limitations, and recommend future works in the conclusion section.

## Materials and methods

The K-complex detection system developed in this work targets the visual detection of K-complexes in EEG waveforms using computer vision techniques in the form of the Faster R-CNN algorithm and deep transfer learning. Thus, building such model relies upon the availability of EEG waveform images and the corresponding K-complex locations. Given a set of EEG recordings and their K-complex scoring (i.e., start and end times of the K-complex), individual images were generated along with separate text files containing the information about the location of the K-complex (i.e., a bounding box expressed as [x, y, width, height] with the x–y coordinates representing the bottom left corner). Two strategies were used to generate the images: (1) One K-complex per image with no repetition of the K-complex. (2) A particular K-complex appear in multiple images by shifting the signal and capturing the image.

The Faster-RCNN object detection algorithm uses the features supplied by a deep neural network backbone, which was implemented using convolutional neural networks (CNNs) and transfer learning. Faster-RCNN taps into one of the feature layers in the CNN model, and uses the output of this layer as features to estimate the location of the bounding boxes with the best overlap in comparison to the ground truth annotated by the experts. Several CNN models can be used, and for each model many options are available for the choice of the feature layer. Four feature extraction CNN models were evaluated in this work.

Once the available data is converted into images with accompanying text files of bounding box locations, the Faster-RCNN algorithm and the CNN model need to be trained, validated, and tested. Several strategies were used for this step: (1) The hold-out method with data pooling from all patients and several data split proportions. (2) Fivefold cross validation. (3) The hold-out method with separate patients used for testing. Moreover, the performance was evaluated using several metrics that reflect the true performance of each model and method.

**Fig. 1:**
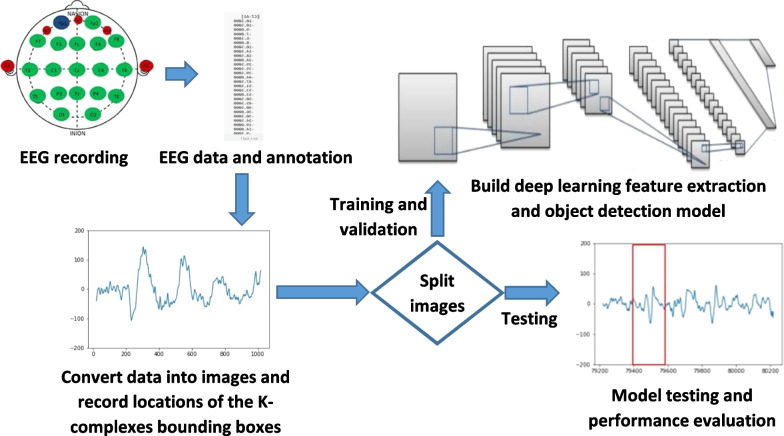
A graphical abstract of the general steps taken to build the K-complex detection model

The general steps followed in this work are shown in Fig. 1. In the next few subsections, we go through each step in detail.

### Dataset

The data used in this study was based on the “Dreams K-complexes database” [27], which is composed of 10 30-min EEG recordings of the central EEG channel extracted from polysomnographic (PSG) sleep recordings. The number of subjects was 10 with each recording corresponding to a single patient only. The EEG was sampled at a rate of 200 Hz, hence each resulting recording contained $30\times 60\times 200= 360{,}000$ data points. The K-complexes were annotated independently by experts with recordings from 1 to 6 were annotated by 2 experts while recording from 7 to 10 was annotated by a single expert.

The EEG signal waveform was divided into multiple images. Each image had a separate text file for the K-complex location, which was expressed using four numbers (i.e., [x, y, width, height]) corresponding to the coordinates of the bottom left corner along with the width and height of the bounding box). The width and height are positive numbers indicating right and up directions, respectively. The images were generated by plotting a 5-s window of the EEG (i.e., at 200 Hz, it is equivalent to $5 \times 200 = 1000$ data points). After that, the window is shifted by 20 points and the data were plotted again. Hence, the difference between two consecutive plots is 0.1 s. Similar segmentation techniques have been reported in the related literature [13]. The total number of frames generated per 30 min of recording is: 30 min $\times$ 60 s $\times$ 10 frames per second = 18,000 frames. Each frame displays a 5-s recording of the EEG signal, which is converted to an image if it contained a K-complex. The total number of resulting images with K-complexes was 10,948.

### K-complex detection model

CNNs are one of the most widely used neural networks types. They are considered one of the most suitable machine learning techniques for discerning image features and discovering spatial and other relationships in visual data [28]. CNNs are composed of a series of convolution and down sampling (i.e., pooling) operations. Different filter sizes control the resolution of the features being considered in the convolution operations. In between these layers, several other operations are conducted to optimize and regulate the network functionality. For example, to avoid overfitting, batch normalization and/or dropout can be used. In addition, various activation functions (e.g., Rectified Linear Unit (ReLU)) can be used to help in learning complicated patterns and in handling nonlinearity. The CNN typically terminates in a fully connected layer or a global pooling operation, which combines features from previous layers to generate the desired output (e.g., classification). The CNN design literature produced a wide range of models that differ in their structure, width, depth, parameters, and regularization. Moreover, they differ in their training efficiency and the methods of updating internal network parameters.

In this work, four CNN models pre-trained using the ImageNet [29] database were used. These were AlexNet [30], VGG19 [31], ResNet-101 [32], and Inceptionv3 [33]. They represent different design philosophies in the CNN literature. AlexNet was one of the early deep CNN designs and consisted of eight layers only due to processing capabilities available at the time. It is a spatial exploitation CNN that improved and fine-tuned many internal operations to reduce overfitting and increase learning efficiency. VGG19 falls under the same spatial exploitation category and consists of 19 layers. The design of the VGG19 network replaces large filters present in AlexNet with a stack of smaller filters. The main premise was that multiple small filters can achieve the same performance as large filters, but with a lower number of parameters and an improved computational complexity. Vastly improved processing powers with the introduction of graphical processing units have spurred the design of more complicated CNNs. As the name suggests, the ResNet-101 consists of 101 layers. Extremely large number of neurons in one layer allows the discovery of more features at a wide range of resolutions but with great overhead. On the other hand, more network depth (i.e., more layers) provides the same benefits with reduced cost [28]. Moreover, several innovations were introduced to reduce the overhead of training very deep networks. In this regard, the ResNet architecture introduced residual learning for more efficient training. The Inceptionv3 model is 48 layers deep and improved on the computational requirements of depth-based networks. This was accomplished by using inception blocks that replaced symmetric large filters with asymmetric small filters, in addition to other modifications [28].

The CNN models act individually as the feature extraction backbone for the object detection algorithm, which was performed using the faster regions with convolutional neural networks (Faster R-CNN) method [5]. This algorithm operates by hypothesizing several object locations using region proposals. Many bounding boxes (i.e., anchor boxes) are proposed as possible locations for the targeted objects. A region proposal network (RPN) drastically reduces the computational overhead of region proposals by sharing the full image convolutional features. Thus, the system structure is composed of a pre-trained CNN (i.e., one of AlexNet, VGG, ResNet101, or Incpetionv3), which serves as a feature extraction network. This feeds to two subnetworks: (1) An RPN that produces possible areas where the K-complexes are likely to be found (i.e., K-complex region proposals). (2) A second subnetwork that predicts the class and bounding box offset of each proposal by using a region of interest (ROI) pooling layer. The designer needs to decide on the optimal location in the CNN to extract the features from. This is best determined empirically or based on design recommendations (e.g., Matlab deep learning toolbox tutorials). The work in this paper followed the Mathworks recommendations for the location of the feature extraction layers and experimented with some other parameters (e.g., positive overlap range). Some of the CNN model properties are shown in Table 1 and further details are provided in the experimental setup section.

**Table 1:** The CNN model properties and the location of the feature extraction layer for K-complex detection

Feature extraction model	Input size	Feature extraction layer	Anchors	Anchor boxes
AlexNet	[227 227 3]	relu5	6	2
VGG19	[224 224 3]	relu5_4	6	2
ResNet-101	[224 224 3]	res4b22_relu	6	2
Inceptionv3	[299 299 3]	mixed7	6	2

### Experimental setup

The evaluation parameters were set as follows: The minimum batch size was set to 2. This was done because the nature of the models, coupled with the number of training images, required large memory space. Higher batches have led to system crashes due to insufficient memory. Unless otherwise stated, the maximum number of epochs was set to 4. Before such epoch was reached, the model training/validation accuracy and loss started to steadily saturate (i.e., no further improvement). The object detector does not require perfect bounding box fits, instead, a positive overlap range needed to be specified for a bounding box to be considered correct. This range was varied from 0.6 to 0.9 in steps of 0.1. It refers to the amount of overlap based on the intersection of union (IoU), which is defined as: $IoU=\frac{Area\, of\, intersection}{Area\, of\, union}$. The data split into training/validation/testing subsets was varied from 60/10/30 to 80/10/10 in steps of 10, with the validation percentage fixed at 10%. The initial learning rate was set to 0.001. The stochastic gradient descent with momentum (SGDM) was used as the solver optimization algorithm. It is a commonly used algorithm for training due to its fast convergence [34], however, other methods are available (e.g., Adaptive Moment Estimation (Adam) optimizer).

The deep learning models were modified, trained, and evaluated using MATLAB R2021a software running on an HP OMEN 30L desktop GT13 with 64 GB RAM, NVIDIA GeForce RTX™ 3080 GPU, Intel Core™ i7-10700K CPU @ 3.80 GHz, and 1TB SSD.

### Performance evaluation metrics

The performance was evaluated using the following metrics, where TP is true positive, FN is false negative, FP is false positive, and FN is false negative:

- Precision (i.e., positive predictive value) defined as: $precision =\frac{ TP }{ TP+FP }$.
- False negative rate (i.e., miss rate) defined as: $miss\ rate= \frac{FN}{TP+FN}$.
- Recall (i.e., sensitivity, hit rate, or true positive rate) defined as: $recall=\frac{TP}{TP+FN}$. It is the complement of the miss rate, and although not explicitly reported, it is used in the precision-recall curve.
- F score, which is defined as the harmonic mean of the Precision and Recall (i.e., $F\ score=\frac{2\times Precision\times Recall}{Precision + Recall}).$
- Precision-recall curve, which plots the value of precision as the class probability threshold is lowered (i.e., higher recall values). A good model will keep the precision high when the recall is increased.
- The log-average miss rate curve, which plots the miss rate against the false positives per image (FPPI) in log scale. Such curves are useful, for example, in each image if you want all the K-complexes to be detected (i.e., less miss rate), then this may lead to more false positives (i.e., part of the signal wrongly detected as K-complex) as a side-effect.
- Average precision (AP) is taken over all images, and the mean average precision (mAP) is taken over all positive overlap thresholds unless otherwise stated.

## Results and discussion

The ability of the various models to detect K-complexes in EEG waveform images was thoroughly evaluated and compared. Table 2 shows the average precision and mean average precision for AlexNet. Using the default Matlab setup (i.e., 60% positive overlap threshold), the model was able to achieve 92.75% to 67.47% AP (81.55% mAP). Moreover, the AP increased as more training data was fed to the model (i.e., 96.24% AP with 80% of the data used for training), which possibly shows more ability to learn without overfitting the data. This trend is also apparent in the other models as well, see Tables 3, 4, and 5. However, the AP degrades as the bounding box acceptable overlap threshold is increased from 60 to 90%. The table shows that there is a large AP drop (about 13%) when the threshold is increased from 80% to 90%, with 60% of the data used for training.

**Table 2:** AlexNet average precision

Data split (%)	60/10/30 (%)	70/10/20 (%)	80/10/10 (%)
Positive overlap threshold			
60	92.75	95.83	96.24
70	85.42	91.15	90.00
80	80.55	81.66	82.49
90	67.47	71.7	81.02
mAP	81.55	85.09	87.44

**Table 3:** VGG19 average precision

Data split (%)	60/10/30 (%)	70/10/20 (%)	80/10/10 (%)
Positive overlap threshold			
60	98.75	99.13	99.44
70	92.94	90.45	94.32
80	90.23	93.67	94.52
90	84.64	89.41	85.21
mAP	91.46	93.17	93.37

**Table 4:** ResNet-101 average precision

Data split (%)	60/10/30 (%)	70/10/20 (%)	80/10/10 (%)
Positive overlap threshold			
60	97.86	98.49	98.68
70	90.77	94.09	97.16
80	83.7	83.69	82.37
90	73.43	79.99	86.53
mAP	86.44	89.07	91.19

**Table 5:** Inceptionv3 average precision

Data split (%)	60/10/30 (%)	70/10/20 (%)	80/10/10 (%)
Positive overlap threshold			
60	97.38	99.8	99.62
70	96.31	97.97	96.57
80	83.78	86.66	91.4
90	91.38	90.55	87.13
mAP	92.21	93.75	93.68

**Fig. 2:**
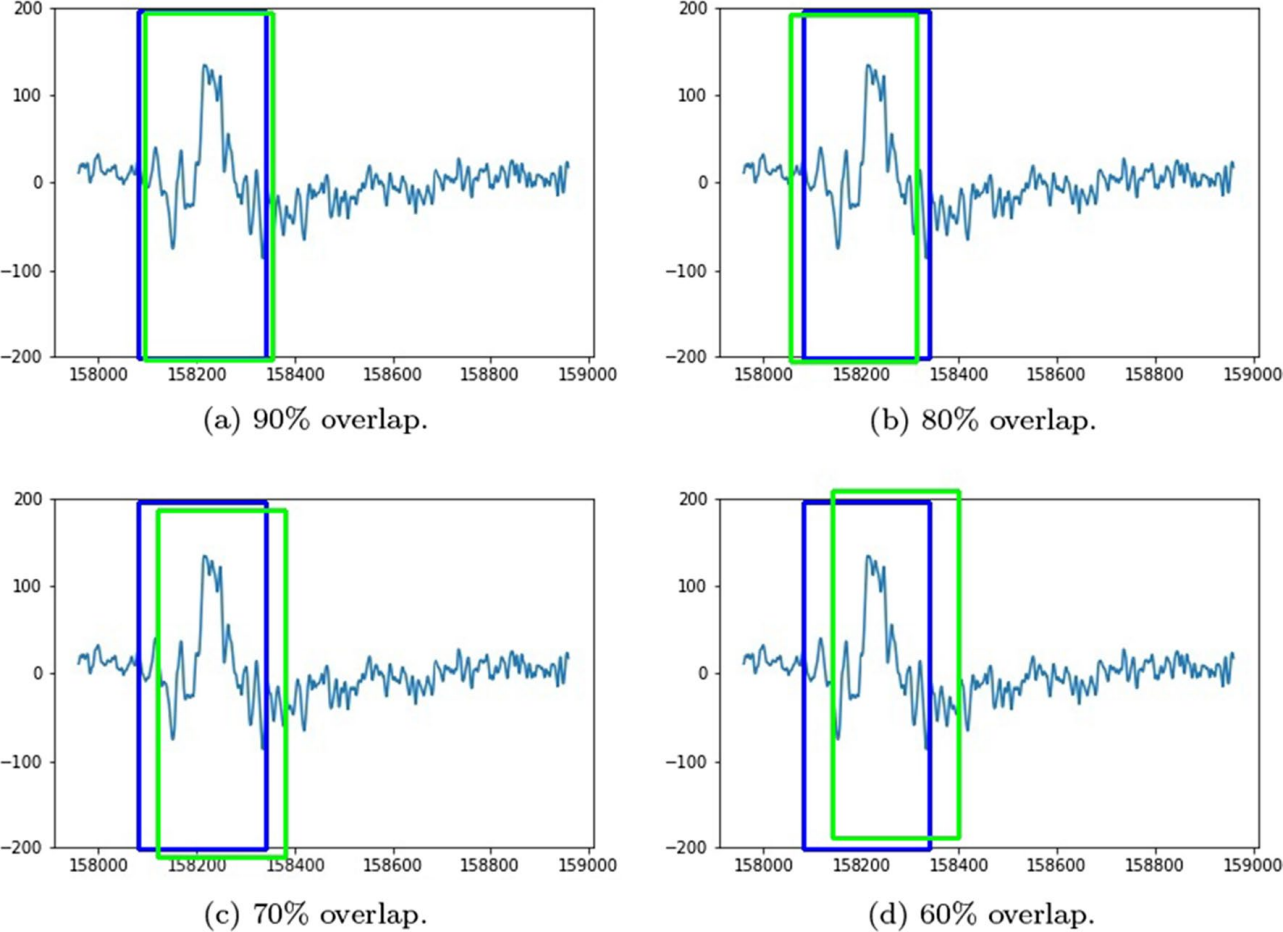
A K-complex with the bounding box as determined by the experts (in blue color) in comparison to multiple overlapping bounding boxes

Such results should be expected as the location of the K-complex is determined by a bounding box, which is compared to the ground truth (i.e., exact location of the bounding box as determined by the experts) for performance evaluation. The 60% positive overlap threshold is the smallest value in the table for the required overlap for a positive detection. This means that this is the lowest par for passing a bounding box as correct. Increasing this threshold means more stringent requirements (i.e., higher passing grade) to consider a bounding box as correct. Nonetheless, a 60% overlap threshold provides good estimates of the K-complex location. Figure 2 provides an indication of the quality of different thresholds for bounding box overlap. In comparison, some related works reported their results for 30% overlap (e.g., Chambon et al. [35, 36]). In addition, the 80/10/10 data split uses 80% of the dataset for training, which is the highest among the other split methods. It is well-known that deep learning models learn better with more data in comparison to traditional machine learning, thus, such results are reasonable [37].

The other three models (Inceptionv3, VGG19, and ResNet-101) perform much better than AlexNet and achieve a maximum AP of 99.62%, 99.44%, and 98.68% respectively. Although the three models follow similar performance trends with increased training data and overlap threshold as AlexNet, the drop is less significant with the Inceptionv3 and VGG19 models. However, the data in Tables 3 and 5 exhibit some discrepancies. For example, there is slight decrease in the accuracy for Incpetionv3 when more data is used for training (e.g., 99.8% AP with 70% data split as compared to 99.62% AP with 80% data split). A similar discrepancy appeared in some limited cases when the positive overlap threshold is increased (e.g., ResNet-101 82.37% AP with 80% threshold and 80% data split compared to 86.53% AP with 80% data split and 90% threshold). This may be caused by model training variations (e.g., random dropout in the deep learning model layers), the availability of more testing data making detection errors less profound in terms of precision, the randomness of the data split (i.e., more difficult to detect cases turn out in the testing set), or in the extreme case an overfitting of the data. However, this should not undermine the excellent AP and mAP results achieved by those models. Moreover, running the models several times should provide more clues into this phenomena, but it requires a long runtime.

**Fig. 3:**
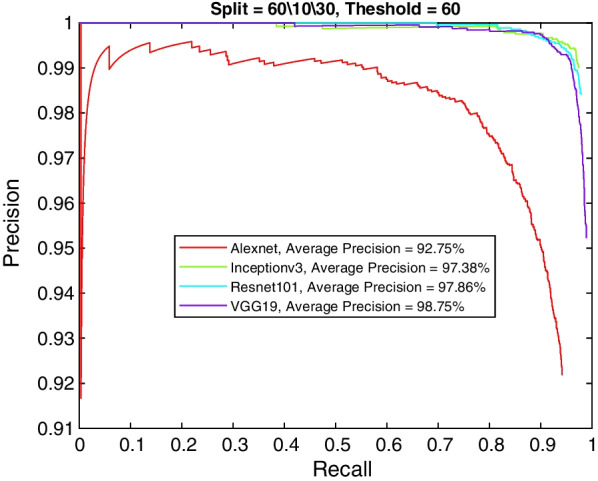
A comparison between all feature extraction models using 60/10/30 data split and 60% positive overlap threshold. The figure plots the precision recall curve

**Fig. 4:**
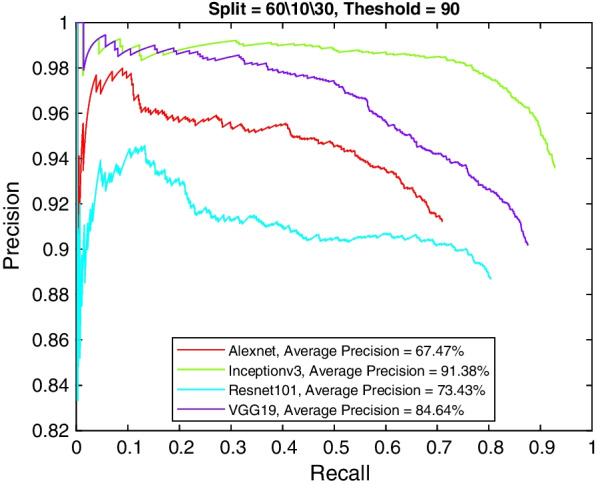
A comparison between all feature extraction models using 60/10/30 data split and 90% positive overlap threshold. The figure plots the precision recall curve

**Fig. 5:**
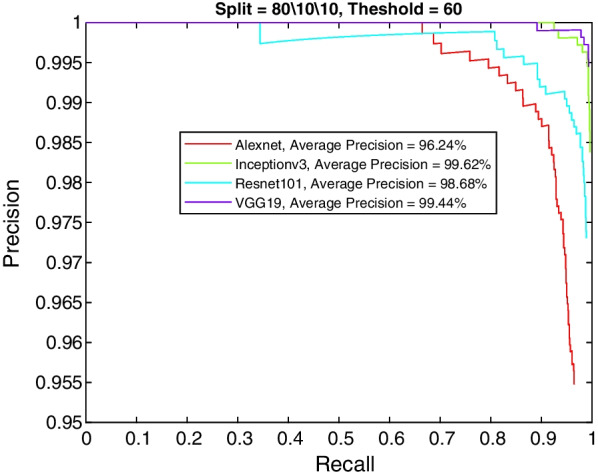
A comparison between all feature extraction models using 80/10/10 data split and 60% positive overlap threshold. The figure plots the precision recall curve

**Fig. 6:**
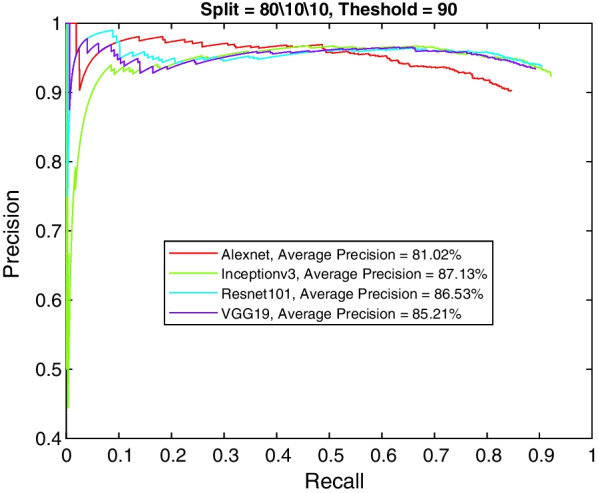
A comparison between all feature extraction models using 80/10/10 data split and 90% positive overlap threshold. The figure plots the precision recall curve

More insight into the K-complex detection performance can be obtained via the precision-recall curve. It gives more information about the relative performance of the various models in relation to the detection class probability. As the recall value is increased (i.e., more bounding boxes are accepted), a good model would have these boxes to be of the correct class or position. In other words, the precision does not drop with higher recall. Figures 3, 4, 5 and 6 show the precision–recall curves for the four models drawn on the same figure for the four corner cases for the data split and penalty threshold (i.e., 60/10/30 split and 60% threshold, 60/30/10 split and 90% threshold, 80/10/10 split and 60% threshold, and 80/10/10 split and 80% threshold). Inceptionv3 and VGG19 are indeed the best performing models.

**Fig. 7:**
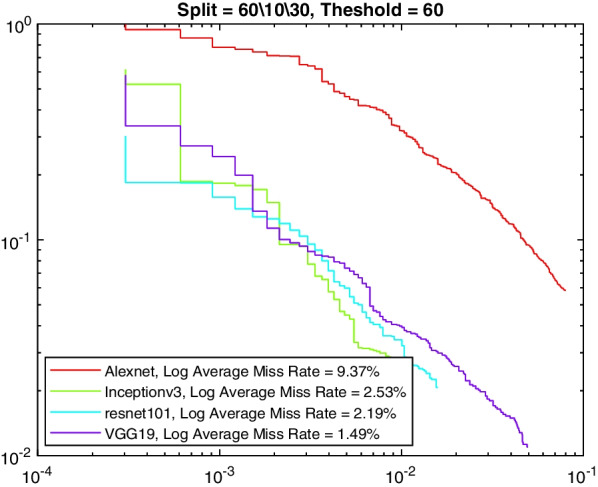
A comparison between all feature extraction models using 60/10/30 data split and 60% positive overlap threshold. The figure plots the log-average miss rate curve

**Fig. 8:**
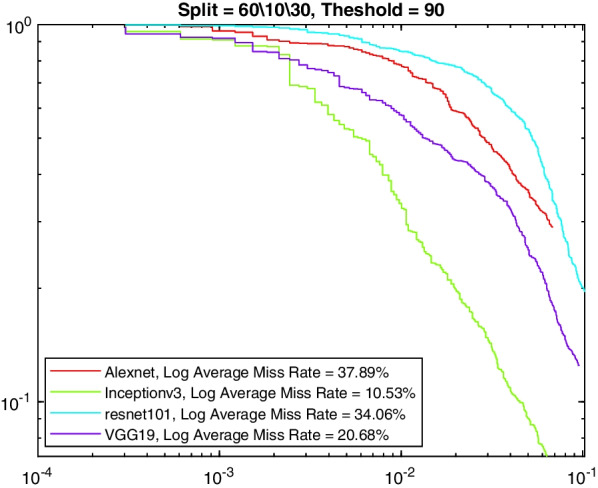
A comparison between all feature extraction models using 60/10/30 data split and 90% positive overlap threshold. The figure plots the log-average miss rate curve

**Fig. 9:**
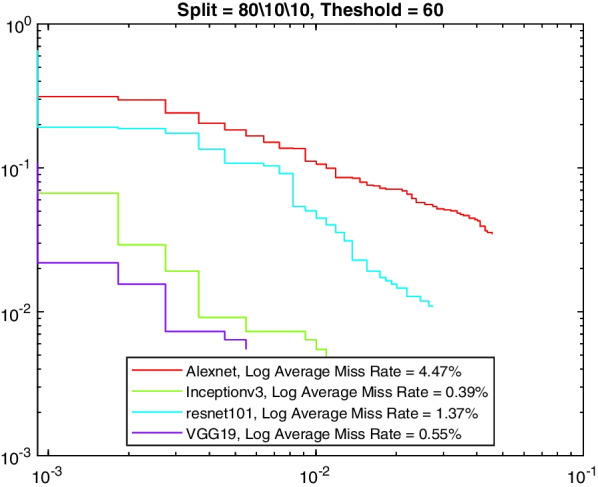
A comparison between all feature extraction models using 80/10/10 data split and 60% positive overlap threshold. The figure plots the log-average miss rate curve

**Fig. 10:**
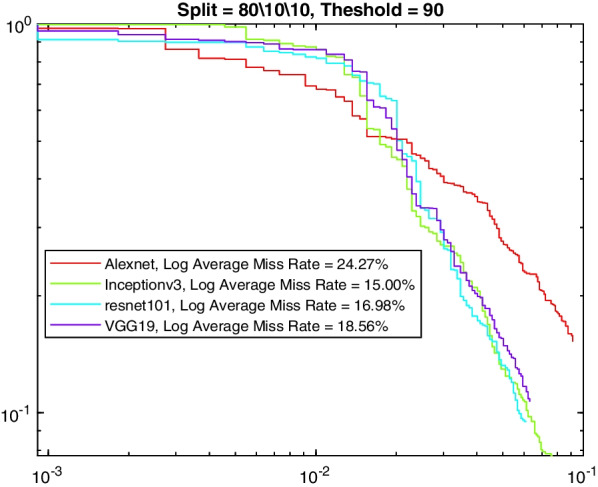
A comparison between all feature extraction models using 80/10/10 data split and 90% positive overlap threshold. The figure plots the log-average miss rate curve

Tables 6, 7, 8, and 9 show the average miss rate for the four models. It is calculated by averaging the miss rate, which evaluates how much K-complexes are missed relative to the number of K-complexes really present, on all the FPPI points. The tables show similar trends and relative performance to the AP measure. Figures 7, 8, 9 and 10 show the log-average miss rate curve for all FPPI points, which follows a similar trend to the precision-recall curves.

**Table 6:** AlexNet average miss rate

Data split (%)	60/10/30 (%)	70/10/20 (%)	80/10/10 (%)
Positive overlap threshold			
60	9.37	5.72	4.47
70	18.09	12.43	12.29
80	25.22	24.22	23.4
90	37.89	33.89	24.27

**Table 7:** Inceptionv3 average miss rate

Data split (%)	60/10/30 (%)	70/10/20 (%)	80/10/10 (%)
Positive overlap threshold			
60	2.53	0.2	0.39
70	4.27	2.44	3.21
80	19.55	14.74	10.15
90	10.53	11.77	15

**Table 8:** VGG19 average miss rate

Data split (%)	60/10/30 (%)	70/10/20 (%)	80/10/10 (%)
Positive overlap threshold			
60	1.49	0.83	0.55
70	7.72	5.22	4.83
80	11.62	12.77	6.22
90	20.68	11.22	18.56

**Table 9:** ResNet-101 average miss rate

Data split (%)	60/10/30 (%)	70/10/20 (%)	80/10/10 (%)
Positive overlap threshold			
60	2.19	1.64	1.37
70	8.6	7.52	3.06
80	19.48	20.56	20.68
90	34.06	23.56	16.98

Table 10 shows the training times for all algorithms using the various data splits. The Inceptionv3 model was by far the slowest requiring 19.63 h for training using 80% of the dataset. The fastest model was AlexNet, which required 2.53 h for training using 80% of the data. However, VGG19 represents an excellent compromise as it requires 4.13 h using 80% of the dataset for training, but achieves comparable accuracy to Inceptionv3. Although these times are large, they are only for training, the testing times were in the order of milliseconds for individual images.

**Table 10:** Average training times (minutes)

Data split	60/10/30	70/10/20	80/10/10
Feature extraction model			
AlexNet	112.5	133.5	152.15
VGG19	189.5	216.3	247.93
ResNet-101	485.7	590.9	660.1
Inceptionv3	818.8	944.4	1178.0

### Cross-validation and testing with separate subjects

The previous results were generated using images of an EEG waveform with K-complexes that display 5 s windows, with a 0.1 s difference between adjacent windows. This may result in very little variations between the different images in the dataset, and consequently lead to data leakage and an inflated good performance. Thus, some of the experiments were repeated with the data modified such that each K-complex in the recording appear in one image only. Hence, a total of 271 images were used for the model training, validation, and testing. Moreover, fivefold cross-validation was used instead of the previous holdout method. Table 11 shows the mean average testing precision using VGG19 for various positive overlap thresholds and training epochs. The mean is taken over the fivefold cross-validation testing subsets. Indeed, the testing precision dropped slightly and required much more training epochs to reach good performance in comparison to the previous evaluation setup.

**Table 11:** Mean average testing precision and F score using VGG19

Training epochs (%)	25 (%)		50 (%)		75 (%)		100 (%)	
Threshold	Precision	F score	Precision	F score	Precision	F score	Precision	F score
60	92.90	91.87	94.8	93.88	95.02	94.03	95.23	95.24
70	79.85	75.99	87.47	85.64	88.66	86.11	92.43	91.11
80	59.39	54.83	84.4	81.55	87.24	85.07	91.10	89.46
90	–	–	72.76	68.3	82.5	79.24	85.22	82.07

The model was trained, validated, and tested using the dataset without repeating any K-complex. The mean is taken over the fivefold cross-validation testing subsets. Threshold is the positive overlap threshold

One more critique of the results relates to the pooling of all recordings from all patients in the dataset, which preceded the split into training, validation, and testing. However, real-life deployment will definitely include subjects not present in the dataset. Thus, to evaluate non-before seen data, separate patients were used in the testing set than in the training and validation sets. Table 12 shows testing precision and F score using VGG19. In this table, the model was trained, validated, and tested using the dataset without repeating any K-complex. Moreover, four subject provided the testing images (45 images) and the remaining 6 subjects were included in the testing (204 images) and validation (22 images). The results show a slight drop in precision in comparison to method of pooling all images from all patients together.

**Table 12:** Testing precision and F-score using VGG19

Training epochs (%)	25 (%)		50 (%)		75 (%)		100 (%)	
Threshold	Precision	F score	Precision	F score	Precision	F score	Precision	F score
60	85.62	84.16	90.85	90.65	92.28	90.72	92.67	91.57
70	76.84	73.04	83.73	82.81	84.43	81.94	85.06	82.96
80	40.24	39.38	78.7	74.63	79.45	76.24	84.09	84.06
90	–	–	65.83	65.62	74.65	71.84	79.44	77.29

The model was trained, validated, and tested using the dataset without repeating any K-complex. The testing subjects were different than the training and validation ones. Threshold is the positive overlap threshold

### Training from scratch versus transfer learning

Deep transfer learning has been shown to be useful and effective in a diverse set of applications from many disciplines (e.g., sentiment analysis, software engineering, human gait analysis, etc.) [38]. However, the excellent performance maybe caused by the network architectural design rather than the transfer (i.e., reuse) of the existing model weights. Thus, we evaluated the role of transfer learning, or lack of, by running fivefold cross validation using the untrained VGG19 network. Table 13 shows the testing precision and F score for a model built from scratch using the same VGG19 architecture without weights. In this table, the network was trained, validated, and tested using the dataset without repeating any K-complex. In comparison to Table 11, the results in Table 13 show a significant drop in precision and little improvement with more training epochs. Such differences in performance can confirm the importance of transfer learning as opposed to training the network from scratch.

**Table 13:** Testing precision and F-score using the untrained VGG19 network architecture

Training epochs (%)	25 (%)		50 (%)		75 (%)		100 (%)	
Threshold	Precision	F score	Precision	F score	Precision	F score	Precision	F score
60	83.88	80.26	84.12	80.98	85	81.31	86.12	82.96
70	78.03	74.61	81.02	77.76	82.98	79.75	84.13	81.43
80	55.55	51.13	75.11	71.88	79.46	76.45	79.67	76.34
90	–	–	60.4	57.4	70.87	67.45	78.5	75.16

The network was trained, validated, and tested using the dataset without repeating any K-complex. Threshold is the positive overlap threshold

### Comparison to the related literature

The application of artificial intelligence has received great attention in the medical literature in general and in the identification of micro-events in EEG signals in particular. Table 14 shows a comparison to the latest results in detecting and locating K-complexes in EEG signals. Chambon et al. [36] designed a dedicated deep neural network architecture to visually detect K-complexes and sleep spindles. Although surpassed the performance in the literature they surveyed, the IoU value of 0.3 (i.e., 30%) is considered small in comparison to our work. Moreover, even with such small IoU, their performance is considerably low in comparison to the precision-recall curves presented in our work. In addition, an extended version of their work failed to achieve a precision value over 80% [36]. Tapia and Estéves [39] proposed recurrent event detector based on a recurrent neural network architecture that the authors designed from scratch. Their model worked in two forms; one uses the EEG signal as a time series and the other employs the spectrogram generated by the continuous wavelet transform. However, their approach follows the traditional path of signal processing and feature extraction. The performance in terms of precision was 84.9% at the very low IoU of 20%.

**Table 14:** Comparison to the state of the art results

Study and years	Method	Results
Dumitrescu et al. 11]	Feature extraction from the Cohen class energy	98.3% Accuracy
Al-Salman et al. 12]	Multi-domain feature extraction	97.7% Accuracy
Al-Salman et al. 13]	Fractal graph features of spectrogram images	97% Accuracy, 96.6% Recall
Oliveira et al. 42]	Multitaper spectral analysis	71.88% Precision, 85.1% recall
Rangan et al. 3]	Fuzzy neural network	87.6% Accuracy 94% recall
Ghanbari and Moradi 40]	Synchrosqueezing Transform	93% Recall
Patti et al. 41]	Pattern matched wavelets	Recall 84%, precision 62%
Chambon et al. 36]	Custom CNN for object detection	Precision < 80% @ IoU = 30%
Tapia and Estéves [39]	Recurrent Event Detector	Precision = 84.9% @ IoU = 20%
This work	Object detection in EEG waveform images	79.4499.44% precision @ IoU = 60%

In terms of performance, Dumitrescu et al. [11] reported comparable results to the ones in this paper, however, their results showed great discrepancies in that the training accuracy was 67.87% as opposed to the testing accuracy of 98.3%, which does not make sense as the artificial intelligence model should be optimized for the former. The studies of Al-Salman et al. [12, 13] performed complex feature extraction using techniques from graph theory and multi-domain features. However, their data preprocessing technique resulted in windows with 80% overlap to adjacent one, and this may lead to data leaking. Moreover, there is no mention in both studies how a detected K-complex is considered correct or wrong in comparison to the ground truth data (i.e., should there be perfect overlap or what percentage is considered correct?). The same argument can be made about the study by Rangan et al. [3]. Other traditional methods as in the works of Ghanbari and Moradi [40] and Patti et al. [41] did not achieve good detection precision.

The work in this paper is different in that it does not rely upon explicit signal processing techniques (e.g., filtration), transformation to other domains (e.g., spectrograms), nor on the quality of the proposed features and the quality of their extraction. Furthermore, the approach follows the natural working of the EEG inspection process by treating the waveform as an image, which enables seamless clinical deployment. Moreover, in comparison to the transfer learning used here, custom CNN designs need thorough evaluation to establish their worthiness in comparison to the well-established models. Also, some of the related works did not precisely define how a correct detection is accepted. Moreover, superior performance in comparison to the literature was achieved over all metrics in most cases.

The present study has some limitations. First, the generation of images from the dataset may have led to data leaking. This is because shifting the waveform by a small amount will result in lookalike images that are easily discovered by the deep learning models. Second, limiting the appearance of K-complexes to one per image resulted in a very small dataset. Deep learning models learn better from larger datasets and produce more stable performance [28]. Third, a larger dataset with more patients will enable more robust evaluation of more diverse EEG waveforms, even if little differences exist across subjects. Fourth, partially existing K-complexes were not handled explicitly by the methods in this work. Partial K-complexes may exist in an EEG recording due to errors in annotation, recording cutoff, or in the image generation procedure (however, such images do not exist in this work). Moreover, no other types of sleep events (e.g., spindles) were considered in the detection and location process. Fifth, Faster R-CNN does not handle windows with no K-complexes [5]. Sixth, it is worthwhile, based on the reported results, to embed the model and other future models in smartphone applications. These should be made available to professionals in order to identify shortcomings and improve the system with feedback and more data (Additional files 1, 2).

## Conclusion

EEG signal analysis is important for the diagnosis of many brain pathologies. This relies on detecting certain events in the signal. The K-complex is one of those features of the EEG waveform. It is used by clinicians in sleep studies and to detect brain abnormalities. However, the graphical inspection of the waveform is tedious, time consuming, and error-prone. Moreover, existing methods in the literature are based on complex signal and image processing techniques that may not be suitable for practical deployment and usability. In addition, they suffer from low accuracy.

Deep learning is gaining great traction in the artificial intelligence literature with many applications spanning various scientific fields especially in classification problems. Given a set of EEG waveform images, this work employed deep transfer learning and Faster R-CNN to determine the location of K-complexes with great precision. Nonetheless, expanding the dataset with more recording from a diverse number of subject should allow more robust evaluation and performance. The increasing real-life deployment of artificial intelligence-enabled devices and applications, and the high robust performance will make it easier to implement K-complex detection applications for usage by clinicians.

## Supplementary Information

**Additional file 1.** System video. This file contains a live video of K-complex detection.

**Additional file 2.** Full results. Full detailed results of the work in this paper.

## Data Availability

The dataset of EEG signals is publicly available from www.doi.org/10.5281/ZENODO.2650142. The images generated from the EEG signals during the current study are available from the corresponding author on reasonable request.

## Abbreviations

EEG: Electroencephalography
CNN: Convolutional neural network
Faster R-CNN: Faster regions with convolutional neural networks
NREM: Non-rapid eye movement
RLS: Restless legs syndrome
OSA: Obstructive sleep apnea
MELM-GRBF: Modified extreme learning machine-generalized radial basis function
Adam: Adaptive Moment Estimation
AI: Artificial intelligence
PSG: Polysomnographic
RPN: Region proposal network
ROI: Region of interest
IoU: Intersection of union
FPPI: False positives per image
AP: Average Precision
mAP: Mean average precision
GPU: Graphical processing units
