# Supplementary material for: Detection of K-complexes in EEG waveform images using faster R-CNN and deep transfer learning
Source: BMC Med Inform Decis Mak. 2022 Nov 17;22:297. doi: 10.1186/s12911-022-02042-x (PMC9673349; doi:10.1186/s12911-022-02042-x)

Figure S1. A comparison between all feature extraction models using 80/10/10 data split and 90% positive overlap threshold. The figure plots the precision recall curve.


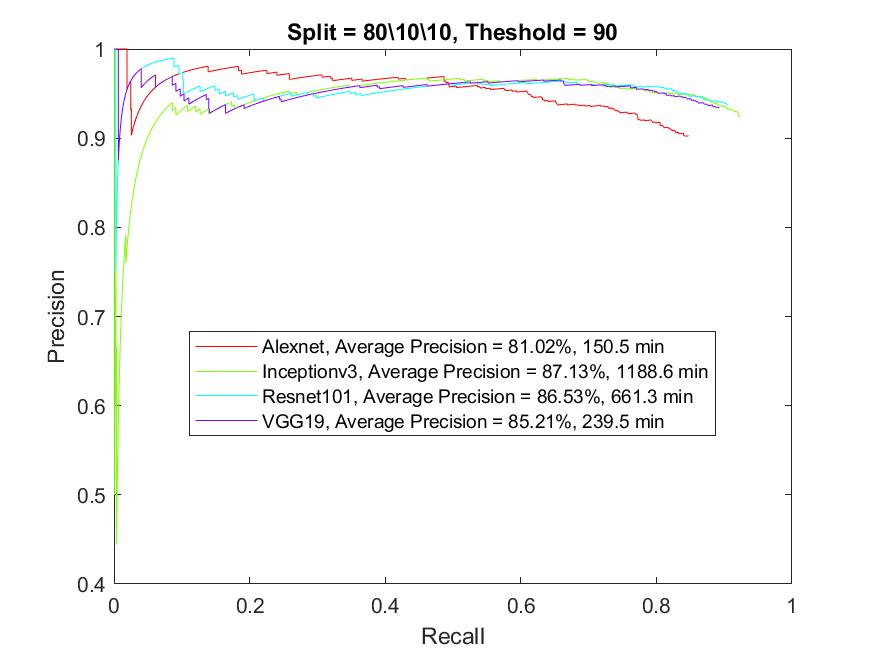


Figure S2. A comparison between all feature extraction models using 80/10/10 data split and 80% positive overlap threshold. The figure plots the precision recall curve.
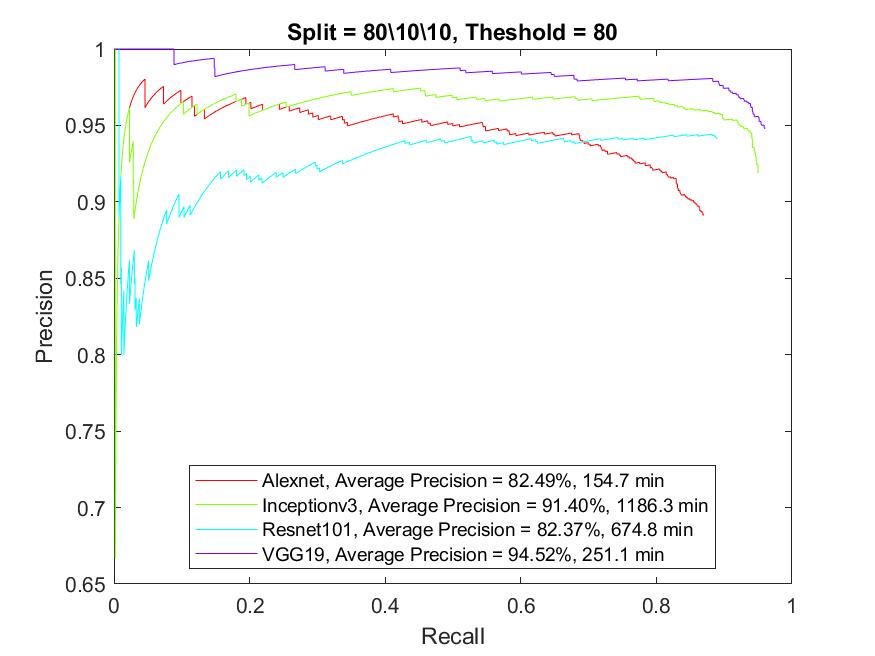


Figure S3. A comparison between all feature extraction models using 80/10/10 data split and 70% positive overlap threshold. The figure plots the precision recall curve.
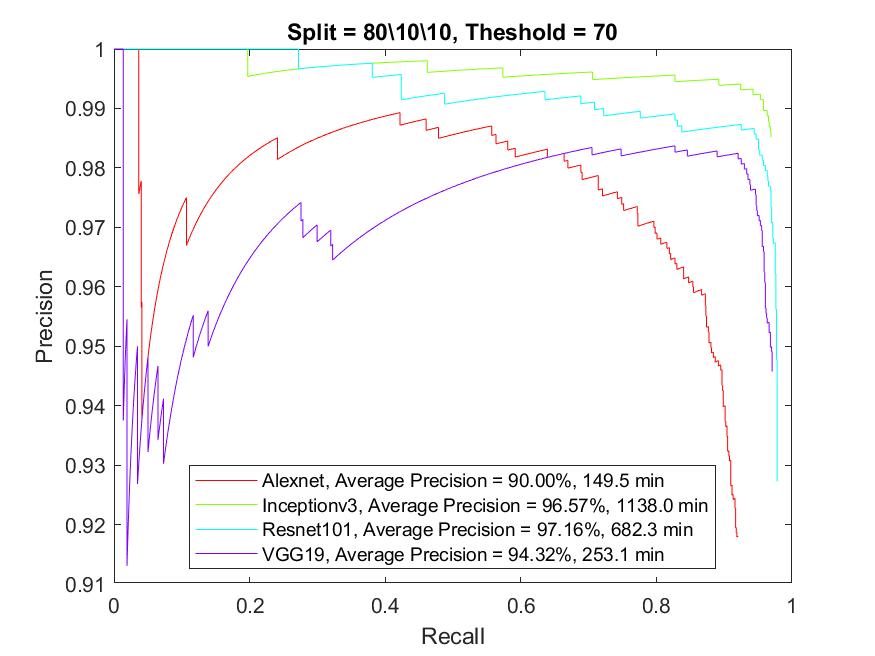


Figure S4. A comparison between all feature extraction models using 80/10/10 data split and 60% positive overlap threshold. The figure plots the precision recall curve.
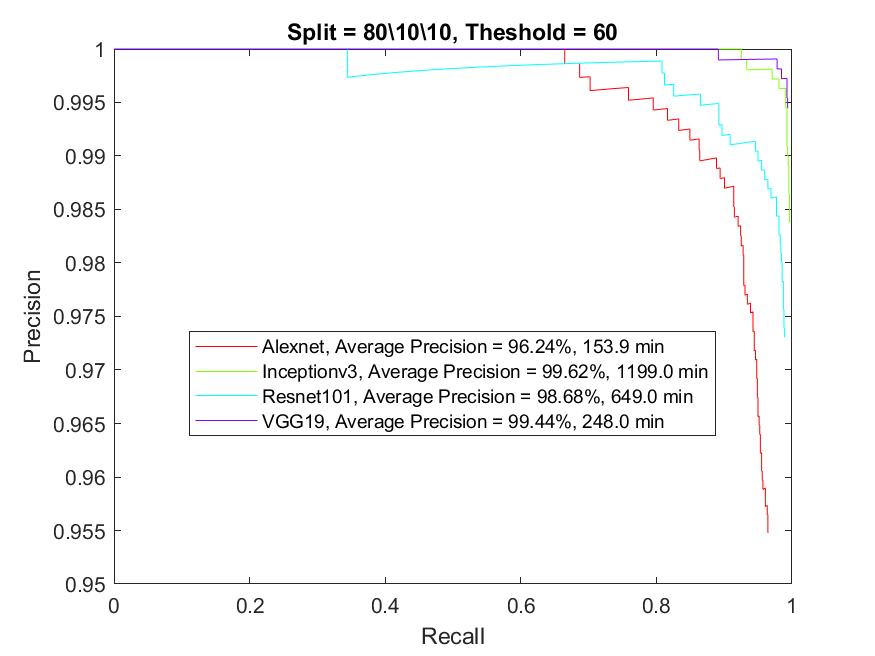


Figure S5. A comparison between all feature extraction models using 70/10/20 data split and 90% positive overlap threshold. The figure plots the precision recall curve.
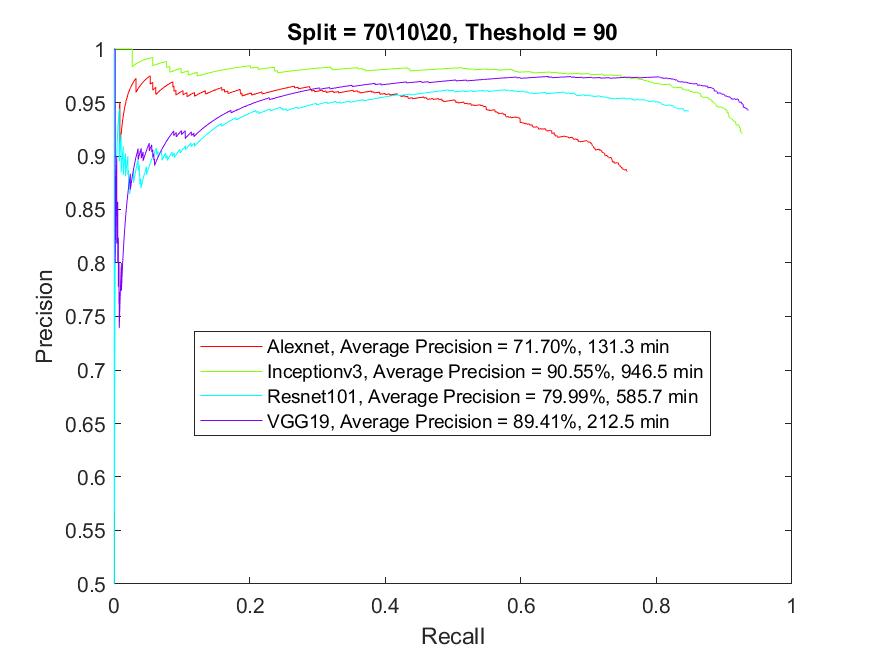


Figure S6. A comparison between all feature extraction models using 70/10/20 data split and 80% positive overlap threshold. The figure plots the precision recall curve.
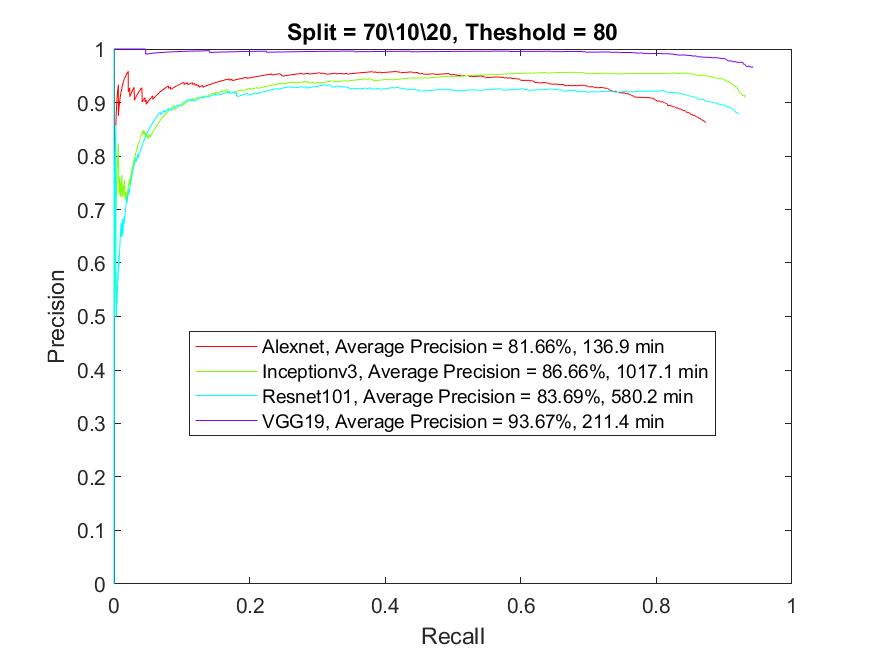


Figure S7. A comparison between all feature extraction models using 70/10/20 data split and 70% positive overlap threshold. The figure plots the precision recall curve.
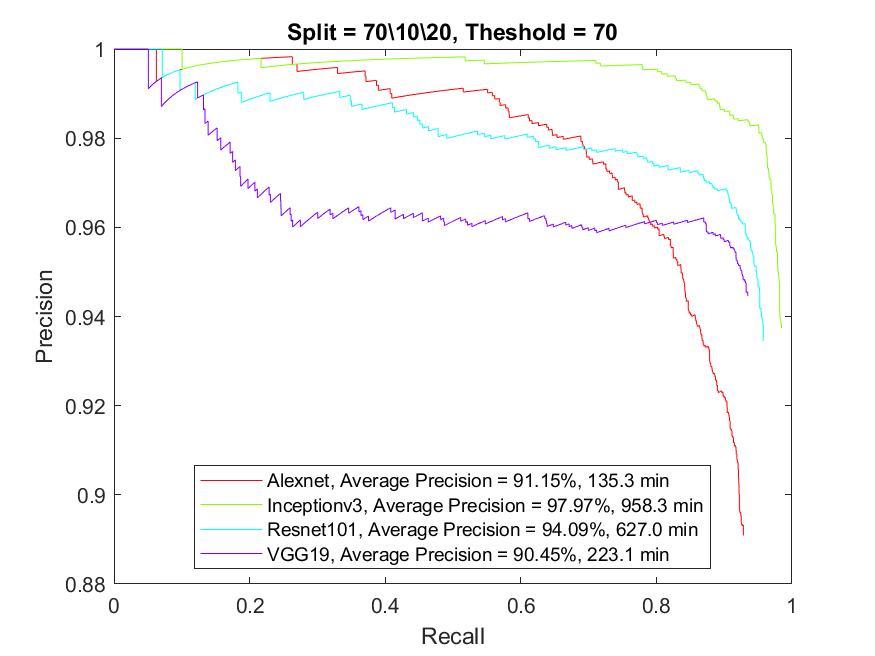


Figure S8. A comparison between all feature extraction models using 70/10/20 data split and 60% positive overlap threshold. The figure plots the precision recall curve.
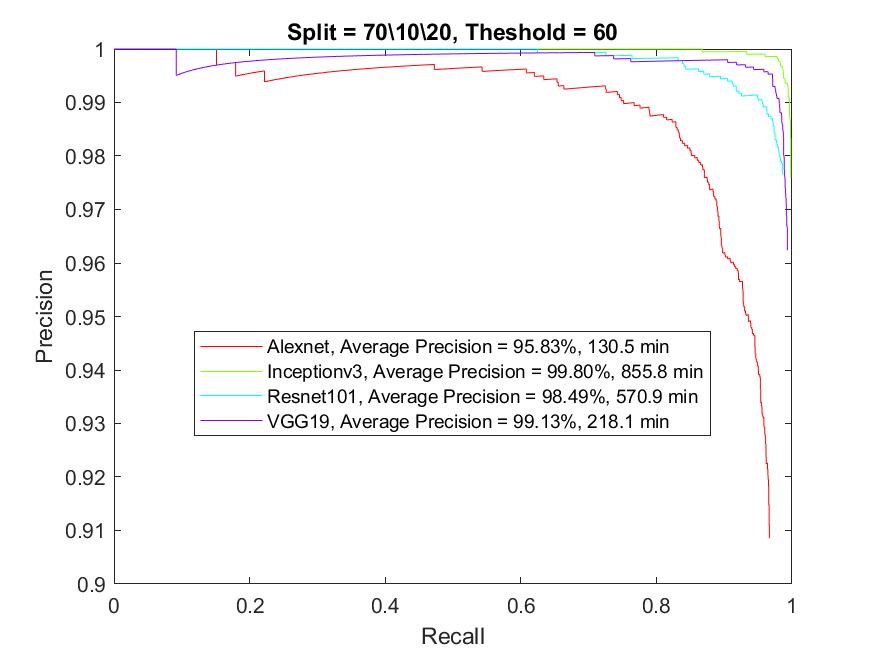


Figure S9. A comparison between all feature extraction models using 60/10/30 data split and 90% positive overlap threshold. The figure plots the precision recall curve.
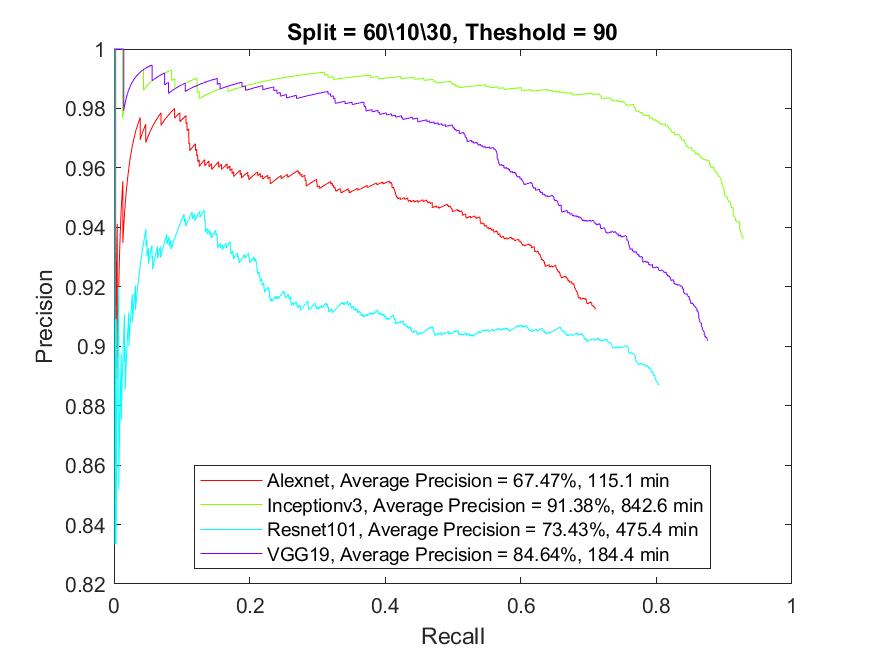


Figure S10. A comparison between all feature extraction models using 60/10/30 data split and 80% positive overlap threshold. The figure plots the precision recall curve.


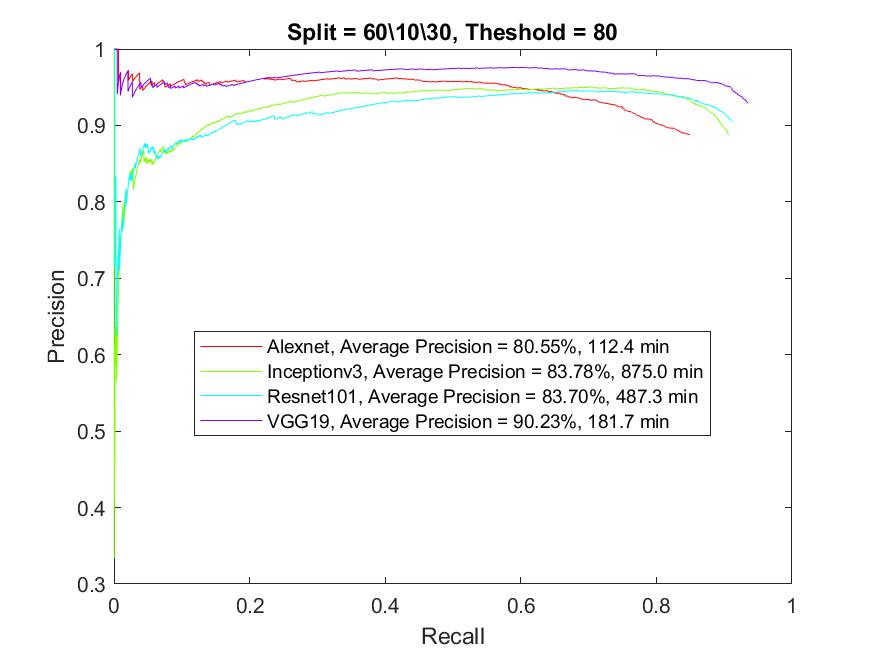
5

Figure S11. A comparison between all feature extraction models using 60/10/30 data split and 70% positive overlap threshold. The figure plots the precision recall curve.
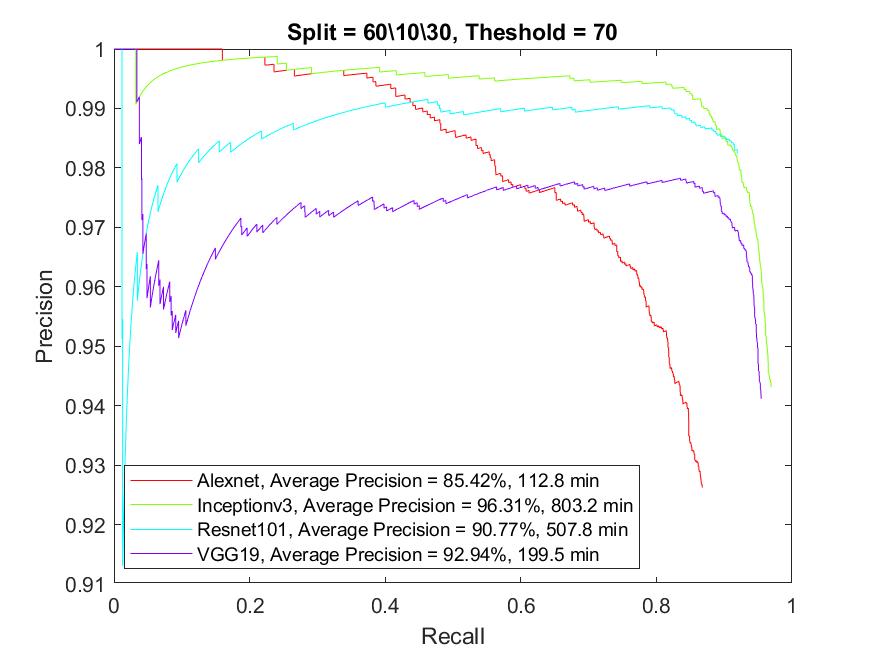


Figure S12. A comparison between all feature extraction models using 60/10/30 data split and 60% positive overlap threshold. The figure plots the precision recall curve.
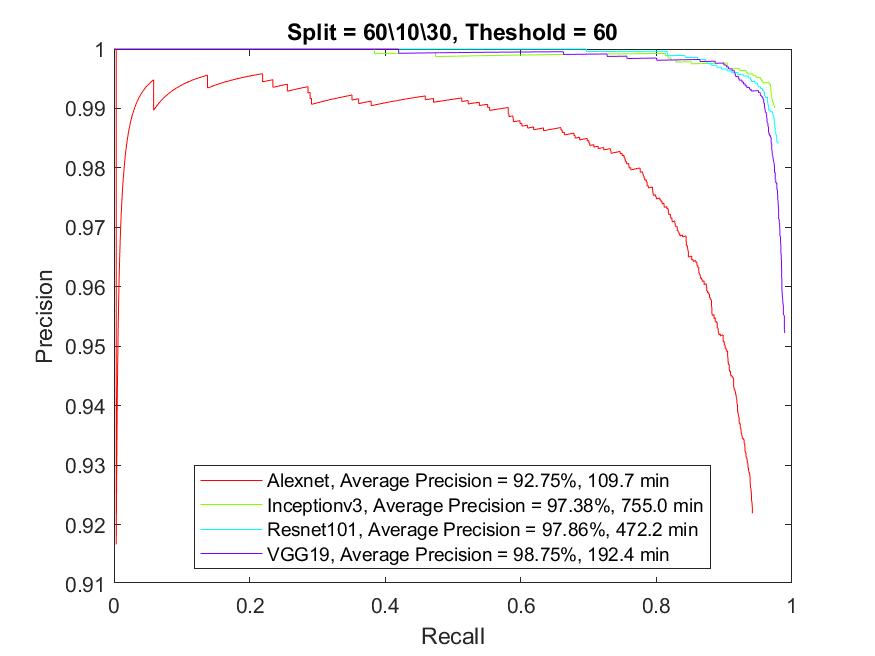


Figure S13. A comparison between all feature extraction models using 80/10/10 data split and 90% positive overlap threshold. The figure plots the log-average miss rate curve.


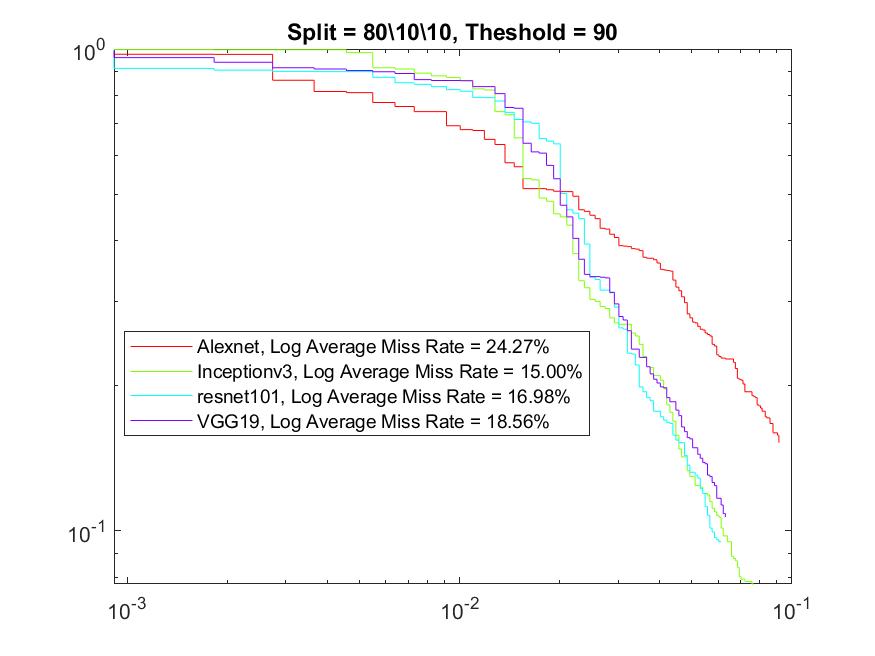


Figure S14. A comparison between all feature extraction models using 80/10/10 data split and 80% positive overlap threshold. The figure plots the log-average miss rate curve.
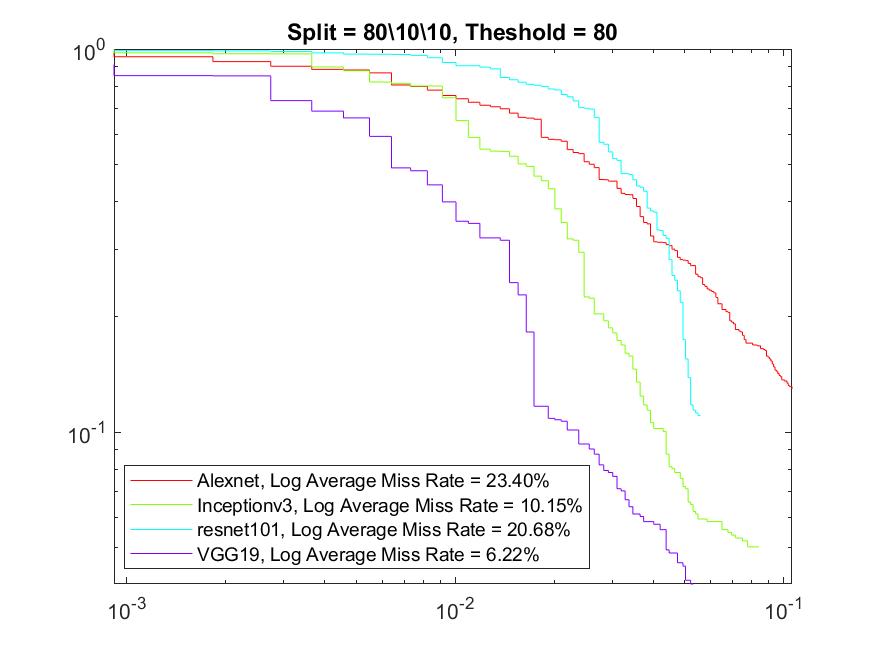


Figure S15. A comparison between all feature extraction models using 80/10/10 data split and 70% positive overlap threshold. The figure plots the log-average miss rate curve.
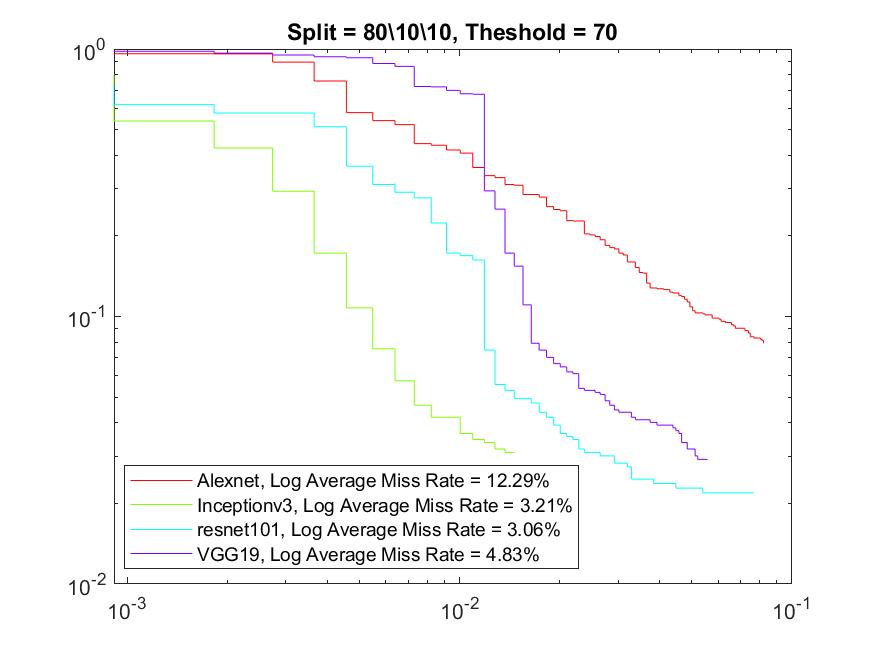


Figure S16. A comparison between all feature extraction models using 80/10/10 data split and 60% positive overlap threshold. The figure plots the log-average miss rate curve.
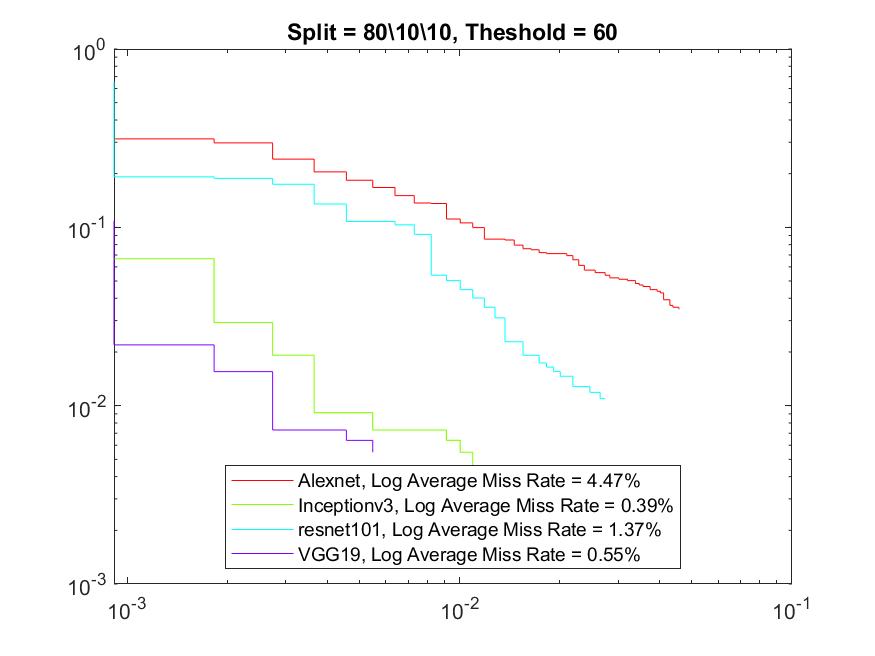


Figure S17. A comparison between all feature extraction models using 70/10/20 data split and 90% positive overlap threshold. The figure plots the log-average miss rate curve.
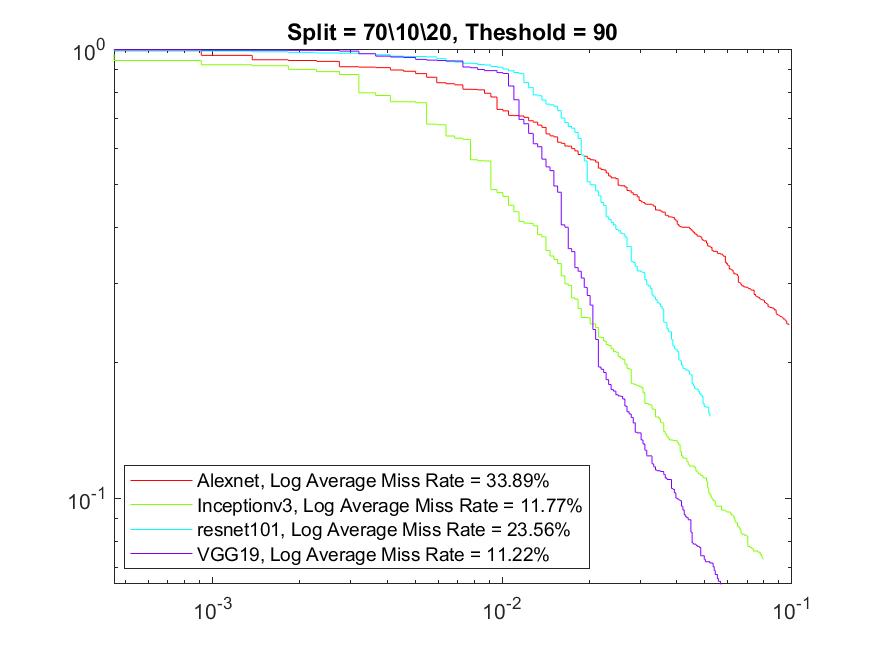


Figure S18. A comparison between all feature extraction models using 70/10/20 data split and 80% positive overlap threshold. The figure plots the log-average miss rate curve.
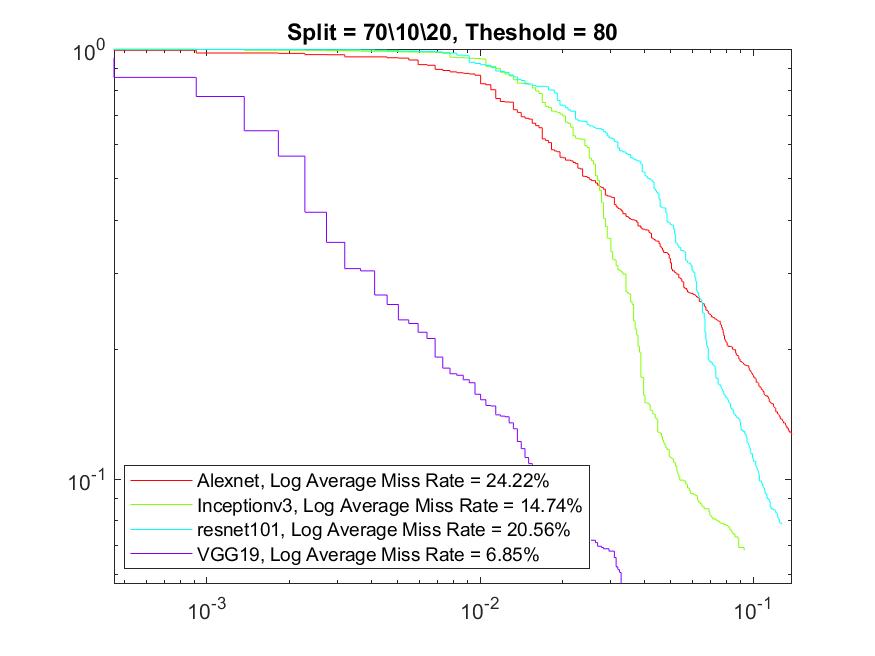


Figure S19. A comparison between all feature extraction models using 70/10/20 data split and 70% positive overlap threshold. The figure plots the log-average miss rate curve.
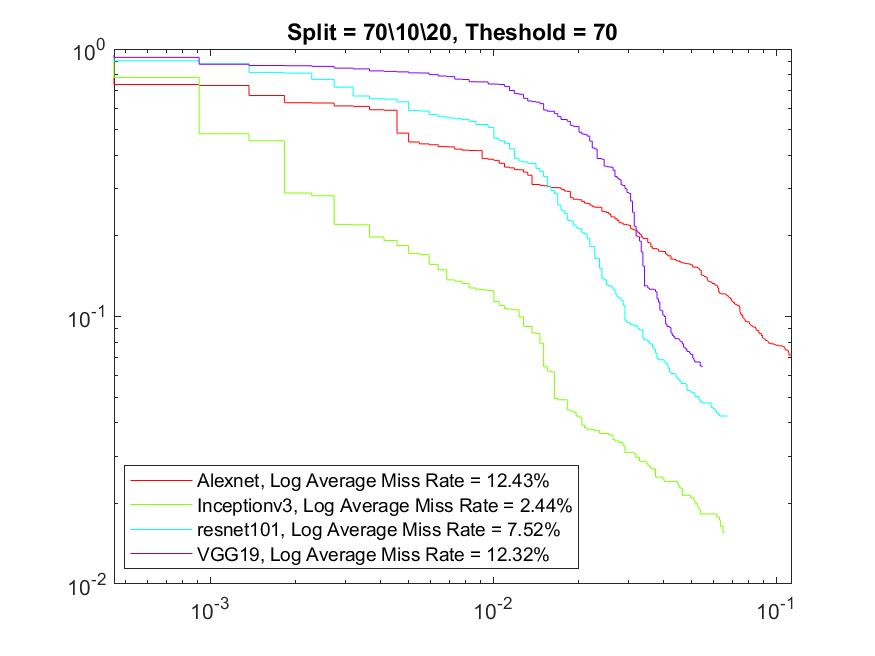


Figure S20. A comparison between all feature extraction models using 70/10/20 data split and 60% positive overlap threshold. The figure plots the log-average miss rate curve.
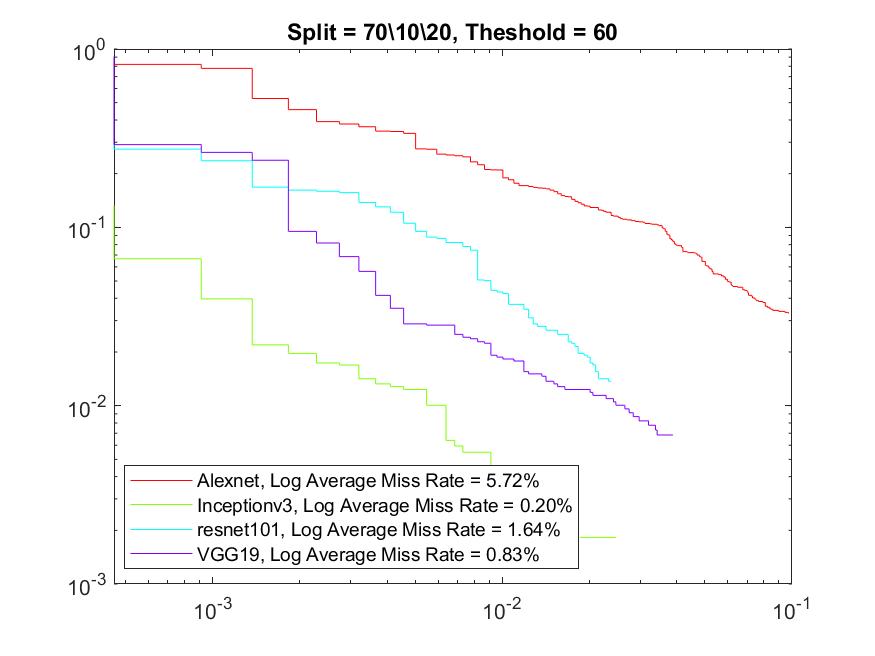


Figure S21. A comparison between all feature extraction models using 60/10/30 data split and 90% positive overlap threshold. The figure plots the log-average miss rate curve.
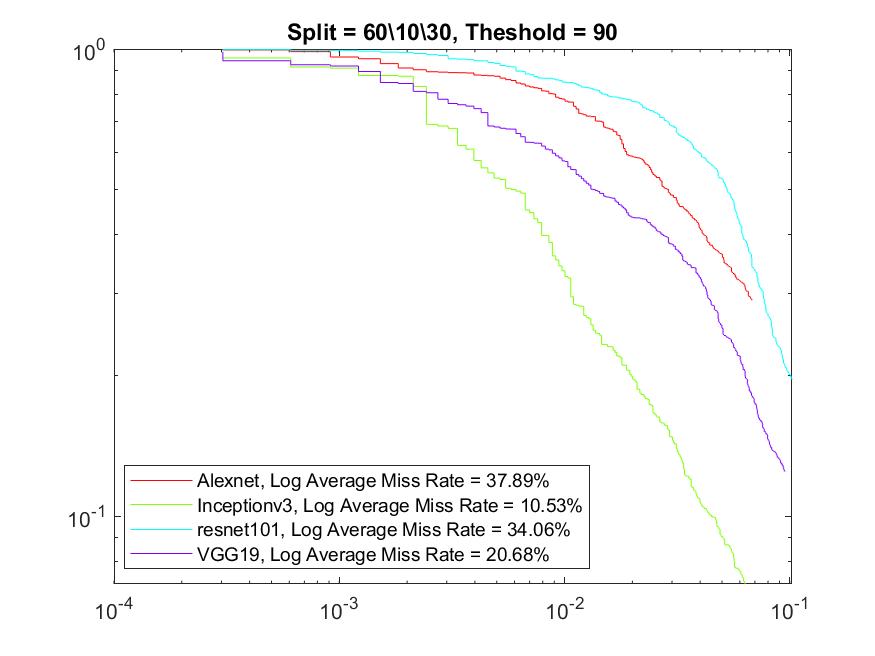


Figure S22. A comparison between all feature extraction models using 60/10/30 data split and 80% positive overlap threshold. The figure plots the log-average miss rate curve.
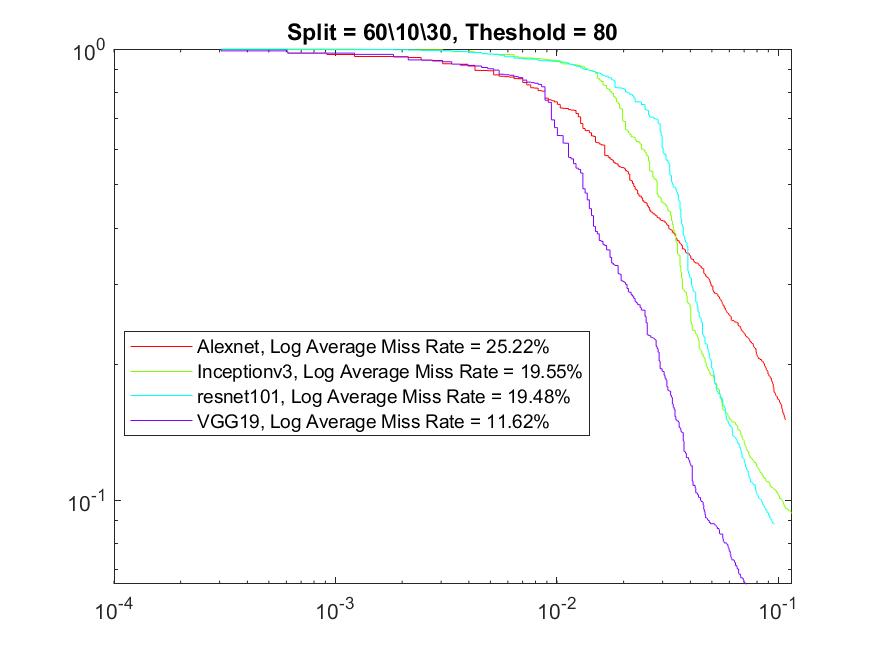


Figure S23. A comparison between all feature extraction models using 60/10/30 data split and 70% positive overlap threshold. The figure plots the log-average miss rate curve.
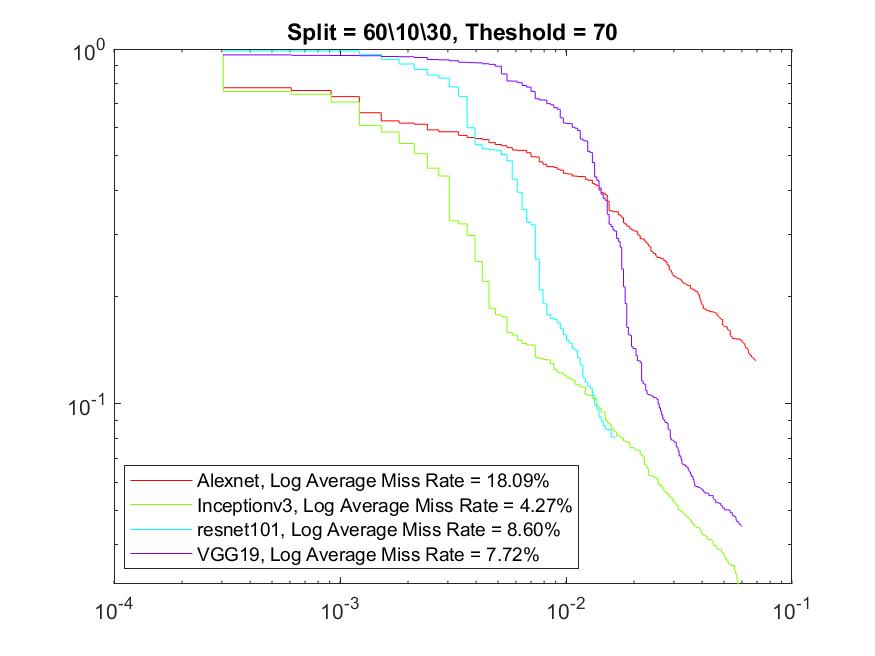


Figure S24. A comparison between all feature extraction models using 60/10/30 data split and 60% positive overlap threshold. The figure plots the log-average miss rate curve.
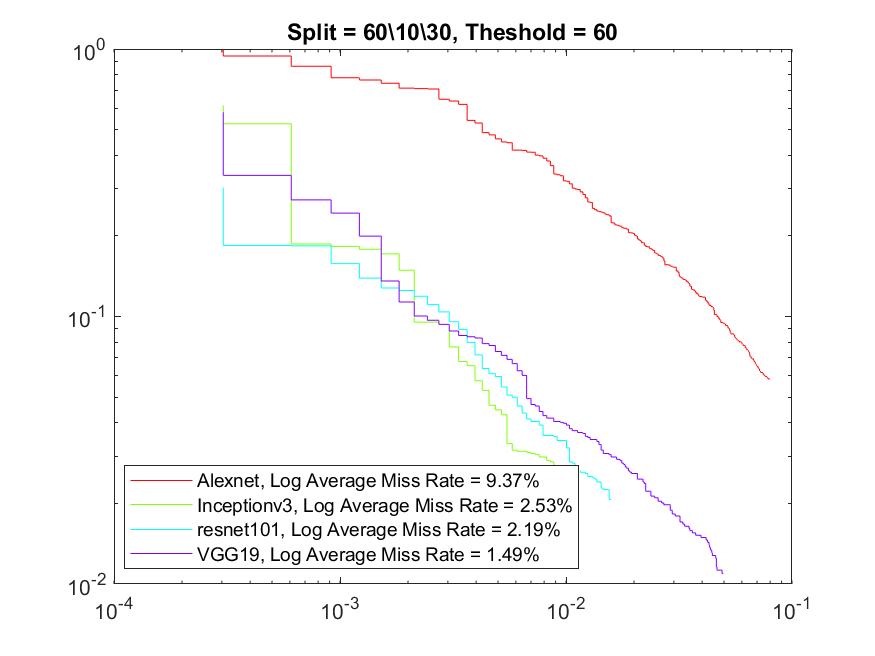

Supplement: Supplementary file 2 — Additional file 2. Full results. Full detailed results of the work in this paper. [file 12911_2022_2042_MOESM2_ESM.docx]
